# Supplementary material for: HIVprotI: an integrated web based platform for prediction and design of HIV proteins inhibitors
Source: J Cheminform. 2018 Mar 9;10:12. doi: 10.1186/s13321-018-0266-y (PMC5845081; doi:10.1186/s13321-018-0266-y)
Supplement: Supplementary file 2 — Additional file 2. Source code of HIVProtI web server. [file 13321_2018_266_MOESM2_ESM.zip › HIVprotI_Source-code/hivprotI/weka-3-6-6/doc/weka/classifiers/trees/j48/SplitCriterion.html]

SplitCriterion


---


|  |  |  |
| --- | --- | --- |
| **Overview** \| **Package** \| **Class** \| **Tree** \| **Deprecated** \| **Index** \| **Help** \| **Weka's home** | |  |
| **PREV CLASS**   **NEXT CLASS** | **FRAMES**    **NO FRAMES**     **All Classes** |  |
| SUMMARY: NESTED \| FIELD \| CONSTR \| METHOD | DETAIL: FIELD \| CONSTR \| METHOD |  |


---


## weka.classifiers.trees.j48 Class SplitCriterion

```
java.lang.Object
  weka.classifiers.trees.j48.SplitCriterion
```

**All Implemented Interfaces:**: java.io.Serializable, RevisionHandler

**Direct Known Subclasses:**: EntropyBasedSplitCrit

---

``` public abstract class SplitCriterion extends java.lang.Object implements java.io.Serializable, RevisionHandler ```

Abstract class for computing splitting criteria
with respect to distributions of class values.

**Version:**
:   $Revision: 1.8 $

**Author:**
:   Eibe Frank (eibe@cs.waikato.ac.nz)

**See Also:**: Serialized Form

---

**Constructor Summary**

`SplitCriterion()`


| **Method Summary** | |
| --- | --- |
| `double` | `splitCritValue(Distribution bags)`             Computes result of splitting criterion for given distribution. |
| `double` | `splitCritValue(Distribution train, Distribution test)`             Computes result of splitting criterion for given training and test distributions. |
| `double` | `splitCritValue(Distribution train, Distribution test, Distribution defC)`             Computes result of splitting criterion for given training and test distributions and given default distribution. |
| `double` | `splitCritValue(Distribution train, Distribution test, int noClassesDefault)`             Computes result of splitting criterion for given training and test distributions and given number of classes. |

**Methods inherited from class java.lang.Object**

`equals, getClass, hashCode, notify, notifyAll, toString, wait, wait, wait`

**Methods inherited from interface weka.core.RevisionHandler**

`getRevision`

**Constructor Detail**

### SplitCriterion

```
public SplitCriterion()
```


**Method Detail**

### splitCritValue

```
public double splitCritValue(Distribution bags)
```

:   Computes result of splitting criterion for given distribution.

    :   **Returns:**: value of splitting criterion. 0 by default

---


### splitCritValue

```
public double splitCritValue(Distribution train,
                             Distribution test)
```

:   Computes result of splitting criterion for given training and
    test distributions.

    :   **Returns:**: value of splitting criterion. 0 by default

---


### splitCritValue

```
public double splitCritValue(Distribution train,
                             Distribution test,
                             int noClassesDefault)
```

:   Computes result of splitting criterion for given training and
    test distributions and given number of classes.

    :   **Returns:**: value of splitting criterion. 0 by default

---


### splitCritValue

```
public double splitCritValue(Distribution train,
                             Distribution test,
                             Distribution defC)
```

:   Computes result of splitting criterion for given training and
    test distributions and given default distribution.

    :   **Returns:**: value of splitting criterion. 0 by default


---


|  |  |  |
| --- | --- | --- |
| **Overview** \| **Package** \| **Class** \| **Tree** \| **Deprecated** \| **Index** \| **Help** \| **Weka's home** | |  |
| **PREV CLASS**   **NEXT CLASS** | **FRAMES**    **NO FRAMES**     **All Classes** |  |
| SUMMARY: NESTED \| FIELD \| CONSTR \| METHOD | DETAIL: FIELD \| CONSTR \| METHOD |  |


---
